# Covid-19 Gastrointestinal symptoms are not a secondary clinical manifestation: the Italian experience 

**Published:** 2020

**Authors:** Roberto Assandri, Alessandro Montanelli

**Affiliations:** 1 *Clinical Investigation Laboratory, Ospedale Maggiore di Crema, Crema, Italy*; 2 *Clinical Investigation Laboratory, Ospedale Bolognini Seriate, Bergamo, Italy*

**To The Editor**

After the December 2019 outbreak of Covid-19 in China, the novel coronavirus infection has quickly spread worldwide and is now considered a global public health crisis. 

In May 2020, a total of 104,291 laboratory-confirmed cases had been documented in Italy, with Lombardy in the northern region of Italian being the major recorder of Covid-19 cases (over 62,000) (Ministry of Health, Civil Protection Department, daily notice).

Lombardy Region reported 79,369 Covid-19 cases, making it a red zone, the eye of the greatest storm ever seen in Italian health history.

Covid-19 causes a clinical syndrome encompassing a wide range of clinical features, from asymptomatic or oligosymptomatic cases to acute respiratory distress and death. 

Early observations in different patient cohorts have indicated that Covid-19 could present with gastrointestinal symptoms (GS), including vomiting, diarrhea, and anorexia/nausea in 3% of patients (1, 2).

In a very recent work, we showed that 10% patients had GS symptoms, confirming the rate described in a Chinese cohort (3). We demonstrated that GS symptoms presented 4.9 days before hospital admission. We also showed that the GS range onset is very wide, with up to 20 days before admission, confirming that GS could be the first signal of Covid-19 infection (3).

Our data also confirmed the importance of including GS symptoms among the spectra of Covid-19 features so as to make early diagnoses and begin appropriate treatments, even in patients without respiratory symptoms. This could be relevant considering the rapid human-to-human transmission of the virus. Viral RNA is now detectable in the stool of patients with suspected Covid-19 (4). Gastrointestinal viral infection and the potential oro-fecal transmission route could persist after viral clearance from the respiratory tract (4). 

In our cohort, GS did not correlate with fever, syncope, or other comorbidities (3). We showed that patients without cough had GS symptoms, suggesting that GS involvement could have an inverse relationship with lung involvement (3).

We also demonstrated that a low lymphocyte count, high C-reactive protein, and liver cytolisis markers (aspartate aminotransferase, lactate dehydrogenase) were significantly associated with death in Covid-19 patients (5,6).

In conclusion, our studies provide strong evidence that GS symptoms were considered the early features of Covid-19 infection, strengthening the need to increase the attention on potential oro-fecal transmission of the virus. 
